# Around the Hospitals

**Published:** 1892-04-23

**Authors:** 


### AROUND THE HOSPITALS.

A little known but much-needed Institution, the **Home and Hospital for Jewish Incurables**, Victoria Park Road, has issued its second report. It has accommodation for twenty-five inmates, but owing to want of funds only fifteen beds have been so far occupied. Patients' payments amount to £192 out of a total income of about £860. The expenditure amounted to £975, so that £200 a year at least is required to carry on the work, and much more will be needed if the ten vacant beds are to be utilised in the interests of Jewish incurables.

The twentieth report of the **Westminster Memorial Cottage Hospital**, Shaftesbury, shows encouraging progress. The Institution relieves 76 in-patients, the average weekly cost per patient, including drugs, being 15s. 6d. The death of the Marchioness of Westminster, the Patroness of the Institution, has caused irreparable loss. We are glad to note that at the annual meeting it was determined not to appoint an assistant nurse for outside cases, but to keep the nursing staff of the hospital distinct from the District Nurse, whom it is proposed to engage to minister to the needs of the poor in Shaftesbury and the immediate neighbourhood.

**The Norfolk and Norwich Hospital** has attracted unusual interest owing to the selection of the Secretary, Mr. Howard J. Collins, for the important post of House Governor to the Birmingham General Hospital. It appears from the report that the "annual subscriptions are still decreasing, though the downward tendency diminished during 1891." Donations, on the other hand, show a corresponding increase. The result of the year's working is that after the expenditure of all the legacies received during the year, and a balance of £248 from 1890, a deficiency of £226 had to be made good out of the invested funds. 1,460 in-patients and 4,885 out-patients were under treatment during 1891. The average cost of each occupied bed was £57 0s. 9d. Mr. B. E. Fletcher has presented the Hospital with a convalescent home, now in course of erection, which will be endowed by the Earl of Leicester. It is interesting to note that the average attendance of members of the Committee at the meetings during the year was upwards of fifteen.

For 119 years the **Leicester Infirmary** has ministered to the poor of the County of Leicester in a markedly efficient manner. It is remarkable that the influence of this infirmary throughout the county has been so great as to prevent the establishment of Cottage Hospitals within its area, a fact which we believe to be unique in the history of English counties. There has been an increase of £1,819 in the income of 1891, as compared with that of the previous year, although we regret to observe that both annual subscriptions and donations show a considerable decrease. Notwithstanding this, the income has exceeded the expenditure by £666, owing to a large increase in legacies. The energetic Mayor, Mr. Alderman Wright, who originated the Children's Hospital now associated with the infirmary, has succeeded in increasing the income for this branch of the work by upwards of £150. It has been decided to erect a new mortuary in a part of the grounds far from the infirmary buildings. Three thousand and thirty-seven in-patients, and 13,897 out-patients were treated, the average cost per week of each in-patient being 18s. 4¾d., the average cost of each out-patient being 2s. Although 37 more in-patients, and 283 more out-patients were treated, the expenditure shows a considerable reduction, the average cost per in-patient having been reduced by no less than 1s. 5d., a fact which should commend the management to the support and confidence of the people of Leicester. We specially commend the publication of a separate account of the income of the Children's Hospital (which account might usefully include the expenditure also), and shall hope that next year the managers of this and of all other important provincial hospitals will publish accounts on the uniform system recently adopted by the Metropolitan Hospital managers.

